# Development of a facile method to compute collagen network pathological anisotropy using AFM imaging

**DOI:** 10.1038/s41598-023-47350-y

**Published:** 2023-11-17

**Authors:** Emilie Khattignavong, Mehrnoosh Neshatian, Mina Vaez, Amaury Guillermin, Josephine T. Tauer, Marianne Odlyha, Nimish Mittal, Svetlana V. Komarova, Hassan Zahouani, Laurent Bozec

**Affiliations:** 1 Faculty of Dentistry, University of Toronto, 124 Edward Street, Toronto, ON M5G 1G6 Canada; 2 UMR 5513, Laboratoire de Tribologie et Dynamique Des Systémes, École Centrale de Lyon-École Nationale d’Ingénieurs de Saint, Université de Lyon, Étienne, France; 3 Shriners Hospital for Children, Montreal, QC Canada; 4 Faculty of Dental Medicine and Oral Health Sciences, McGill University, Montreal, QC Canada; 5 School of Biological Science, Birkbeck College, University of London, London, UK; 6 Division of Physical Medicine and Rehabilitation, Temerty Faculty of Medicine, University of Toronto, Toronto, ON Canada

**Keywords:** Biophysics, Computational biology and bioinformatics, Structural biology

## Abstract

Type I collagen, a fundamental extracellular matrix (ECM) component, is pivotal in maintaining tissue integrity and strength. It is also the most prevalent fibrous biopolymer within the ECM, ubiquitous in mammalian organisms. This structural protein provides essential mechanical stability and resilience to various tissues, including tendons, ligaments, skin, bone, and dentin. Collagen has been structurally investigated for several decades, and variation to its ultrastructure by histology has been associated with several pathological conditions. The current study addresses a critical challenge in the field of collagen research by providing a novel method for studying collagen fibril morphology at the nanoscale. It offers a computational approach to quantifying collagen properties, enabling a deeper understanding of how collagen type I can be affected by pathological conditions. The application of Fast Fourier Transform (FFT) coupled with Atomic Force Microscope (AFM) imaging distinguishes not only healthy and diseased skin but also holds potential for automated diagnosis of connective tissue disorders (CTDs), contributing to both clinical diagnostics and fundamental research in this area. Here we studied the changes in the structural parameters of collagen fibrils in Ehlers Danlos Syndrome (EDS). We have used skin extracted from genetically mutant mice that exhibit EDS phenotype as our model system (*Col1a1*^*Jrt/*+^ mice). The collagen fibrils were analyzed by AFM based descriptive-structural parameters, coupled with a 2D Fast Fourier Transform(2D-FFT) approach that automated the analysis of AFM images. In addition, each sample was characterized based on its FFT and power spectral density. Our qualitative data showed morphological differences in collagen fibril clarity (clearness of the collagen fibril edge with their neighbouring fibri), D-banding, orientation, and linearity. We have also demonstrated that FFT could be a new tool for distinguishing healthy from tissues with CTDs by measuring the disorganization of fibrils in the matrix. We have also employed FFT to reveal the orientations of the collagen fibrils, providing clinically relevant phenotypic information on their organization and anisotropy. The result of this study can be used to develop a new automated tool for better diagnosis of CTDs.

## Introduction

The skin is the largest organ in the human body, serving as a protective barrier between the internal organs and the external environment and offering the first defense against environmental stressors such as UV radiation, pollution, and pathogens. The skin dermal component comprises mostly type-I collagen, secreted by dermal fibroblasts. Other types of collagens, such as collagen Type III and, more rarely, Type V, can also be found in the dermis^1^ .The dermis is 2–3 mm thick, making up most of the thickness of our skin (80% of which is composed of type-I collagen), and is composed of two layers: the papillary dermis (a thin network mesh of type-III collagen) and the reticular dermis (thick bundles of collagen type-I fbrils). Other major components of the dermal extracellular matrix (ECM) include elastic fbrils (elastin), which provide mechanical elasticity, allowing responsiveness to physical loading^2^; proteoglycans, decorin, and versican, with a protein core and sulfated glycosaminoglycan (GAG) chains which are highly hydrophilic and provide water retention properties to the ECM; and hyaluronic acid (HLA), a non-sulfated GAG chain which, due to its hydrophilicity, also has a great capacity to bind with water, providing hydration and water transport to the dermis. Of all 28 types of collagens identified to date, Type I Collagen is by far the most prevalent and studied. In Type I collagen, each molecule comprises three polypeptide chains twisted together, forming a well-defined triple helix^3–5^, known as procollagen. The successful cleavage of N- and C-propeptides^6,7^ from the procollagen molecules causes the spontaneous self-assembly of large supramolecular structures in an entropy-driven process known as fibrillogenesis. These newly formed collagen fibrils are stabilized initially by weak dispersive and hydrogen interactions only, while the covalent bonds are established at a later stage. The fibrils are cylindrical and have a diameter ranging from 10 up to 500 nm with a periodically banded pattern of ∼67 nm known as D-banding, characterized by the presence of overlap and gap regions along the long-axis of the fibril^8,9^ as found in normal bone, dentin, skin, and tendon tissue. The D-banding in collagen refers to a characteristic repeating pattern of dark and light bands observed in electron micrographs of collagen fibrils^10^ when viewed under certain conditions. These bands result from how collagen molecules are organized within the collagen fibrils, which are the structural units of collagen fibrils according to the^11^ our more recent collagen rope model^12^. While the collagen structure is well-defined and conserved across tissues, any alterations in the collagen fibrils' hierarchal assembly can lead to several pathological conditions, such as osteogenesis imperfecta, scurvy, and EDS^13–16^. Exploring any alterations in the collagen fibrils’ morphology is non-trivial as it requires using high-resolution microscopes such as Transmission Electron Microscopes or Atomic Force Microscope^17,18^. Even though it is plausible to observe fibril characteristics such as the D-banding periodicity using these microscopy techniques, it can become challenging to assert whether these changes in the fibril morphology can be directly related to specific pathology^19^, especially since there are no gold standard reference techniques to confirm (clinically) these structural findings at the collagen fibrils level. That is why limited work has been done to associate collagen phenotype at the nanoscale with known pathologies such as fibrosis^20^ scleroderma^19^, and even natural aging From a dermal matrix and fibrils analysis point of view, several automated image-processing techniques have also been used to quantify collagen fibril orientation and organization^21,22^, using techniques such as confocal, second harmonic generation, scanning electron microscopy, and even atomic force microscopy^23–27^. The native organization of collagen fibrils in tissue such as skin^28–30^ and the repetitive D-Banding periodicity on the surface of native collagen fibrils are well-suited for computational analysis using mathematical approaches. FFT is an automated image processing technique used to study fibril properties such as contrast, appearance, alignment, orientation, and angular distribution on images acquired. The Fourier method gives the ability to acquire information on the images utilizing the Fourier transform, filtering, calculating structural parameters of collagen fibrils, and quantifying them^22^ and does not require a prior in-depth understanding of the collagen structural morphology expected as a function of the pathology studied.

Here we have used FFT coupled with AFM imaging to study collagen fibrils isotropy and angular distribution as a computational method for distinguishing healthy and EDS-affected skin. While AFM has previously been used to study the collagen fibrils'^19,20,31^ organization, coupling this imaging modality with a computational approach provides more information on the fibrils' organization and alignment. Furthermore, this technique is no longer limited to local measurements. Ultimately, using this automated image processing method, we could distinguish between healthy and diseased skin using AFM images. The developed method can be used for the automated diagnosis of CTDs. Additionally, the result of this study will allow for a better understanding of CTDs and how they affect the morphology of collagen fibrils, which may help scientists and clinicians explore the impact of CTDs and their treatment at the nanoscale.

## Materials and methods

### Skin samples

Murine skin biopsies were acquired from female *Col1a1*^*Jrt/*+^ mice and their respective wildtype littermates. *Col1a1*^*Jrt/*+^ mice underly a splice site mutation in exon 9 of the *COL1A1* gene leading to an 18-amino acid deletion in the collagen type I a1 chain . *Col1a1*^*Jrt/*+^ mice are smaller in size and demonstrate beside an osteopenic phenotype a skin hyper elasticity conform with EDS phenotype. *Col1a1*^*Jrt/*+^ breeding colony is maintained at the Animal Care Facility of the Shriners Hospitals for Children-Canad. All experiments were approved by the Animal Care Committee of McGill University, complied with ARRIVE guidelines^32^, and conformed to the ethical guidelines of the Canadian Council on Animal Care (Protocol No.7127). Four 2mm-by-2mm dorsal skin sections were acquired from 14-week-old wildtype and *Col1a1*^*Jrt/*+^ mice (n = 2 each). The skin tissues were frozen in optimal cutting temperature resin (OCT) (VWR- Richmond, IL. USA). Serial tissue sections, 10μm thick, were made longitudinally through the epidermis and papillary dermis cryostat (Leica Microsystems CM1850, Germany). Each 10μm section was placed onto a Superfrost™ Plus Microscope Slides(Fisherbrand, USA). For AFM imaging, OCT was removed by immersing microscope slides in 37°C double-distilled, deionized water for 5 min, followed by ethanol dehydration (50, 70, 90, and 100%).

### AFM imaging

Topographical and deflection images (5 × 5 µm^2^, 512 × 512pixel) were acquired using a Nano-wizard 4 Bioscience (Bruker, Germany) operated in contact mode under ambient conditions. MSLN-10-C probes (Bruker, Germany) were used. All images were acquired directly on the histological section within the reticular dermis area of the murine skin biopsies. Seven areas were selected randomly in the region of interest, and image acquisition was optimized for each location where the probe landed. All image analysis was performed on the deflection images recorded using JPK data processing software (version 6.3.5). as the D-banding is readily apparent on this type of image without further processing such as flattening. Avoiding further image processing is essential to ensure that we do not “modify” the appearance of the collagen matrix recorded.

## Fast fourier transform and power-spectrum density calculation

All collagen images were computed in MATLAB (version 9.12.0.1884302, The MathWorks, Inc., Natick, Massachusetts, United States). Two-dimensional Fast Fourier Transform (2D FFT) was performed for each AFM image taken. Images were converted to the frequency domain by means of MATLAB's fft2 function, which returns the two-dimensional discrete Fourier transform of an image size M × N pixels using a fast Fourier transform algorithm (Eq. 1):

1 $$\mathrm{F}\left(\mathrm{u},\mathrm{v}\right)=\sum_{\mathrm{x}=0}^{\mathrm{M}-1}\sum_{\mathrm{y}=0}^{\mathrm{N}-1}\mathrm{f}(\mathrm{x},\mathrm{y}){\mathrm{e}}^{-\mathrm{i}2\uppi \left(\frac{\mathrm{ux}}{\mathrm{M}}+\frac{\mathrm{vy}}{\mathrm{N}}\right)}$$

where x and y represent the spatial coordinates of the image, u and v are the frequency components in the x and y directions, and $\mathrm{i}= \sqrt{-1}$ ^24^. The frequencies represent changes in the pixel intensities. While the Fourier Transform is often complex and contains real parts *Re*(u,v) and imaginary parts *Im*(u,v), the conversion of the Fourier transform into a power spectrum without imaginary parts generates the PSD (Eq. 2). The PSD corresponds to the square of the module of the FFT. Thus, after squaring the FFT, we can use the PSD to directly characterize the spatial frequencies related to repeating structural domains in collagen, such as the D-banding.

2 $${\text{P}}\left( {{\text{u}},{\text{v}}} \right) = { }\left| {{\text{F}}\left( {{\text{u}},{\text{v}}} \right)} \right|^{2} = {\text{Re}}^{2} \left( {{\text{u}},{\text{v}}} \right) + {\text{Im}}^{2} \left( {{\text{u}},{\text{v}}} \right)$$

where u and v are the frequencies in the x and y directions, F(u,v) is the Fast Fourier Transform, *Re*(u,v) is the real parts, and *Im*(u,v) is the imaginary parts^24,26^.

### D-banding measurements

**Figure 1:**
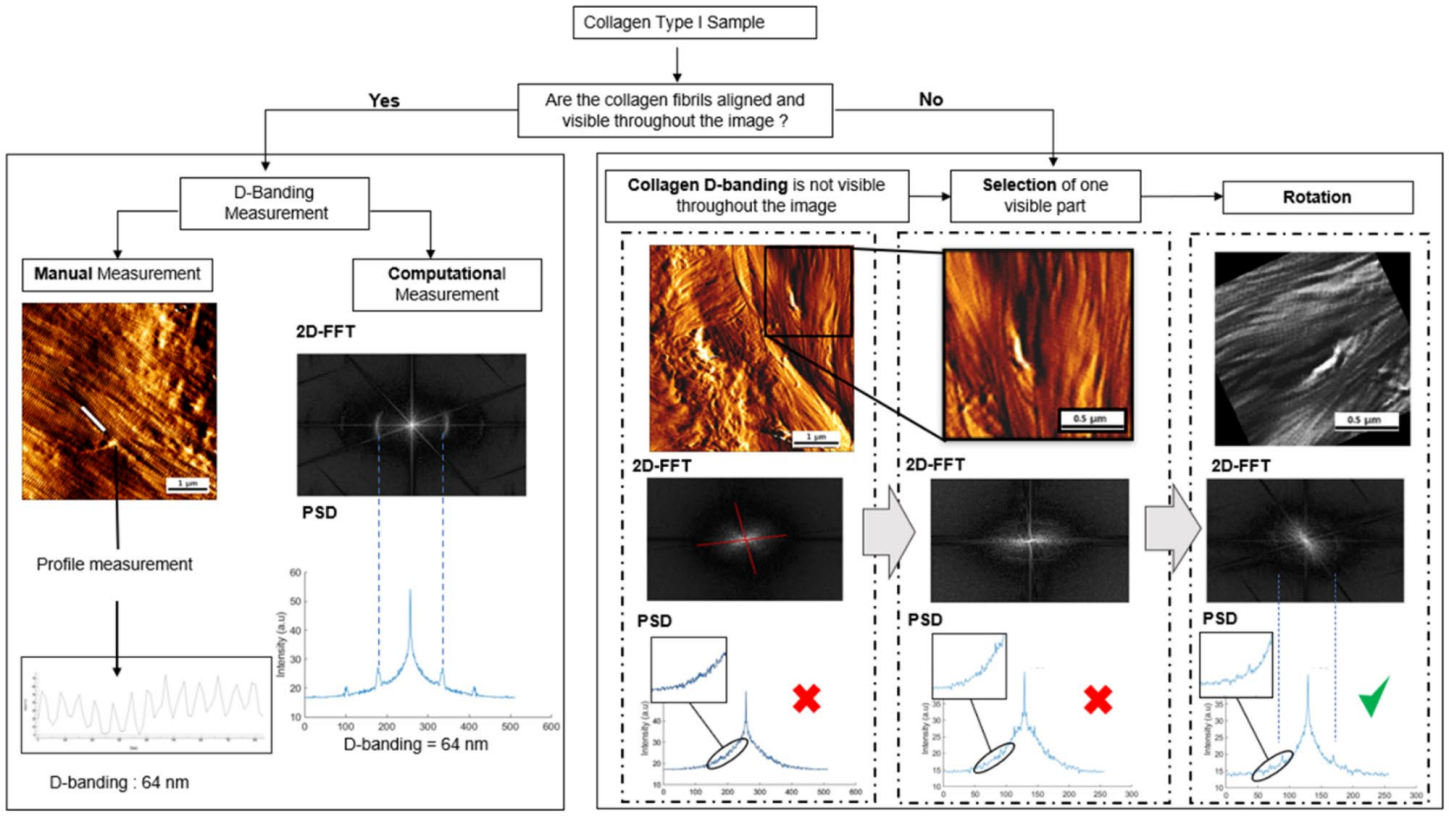
Workflow used to calculate the D-banding periodicity using manual or computational approaches. The computational approach relies on calculating the 2D FFT and rotating the AFM image to maximize the amplitude of the fundamental and first harmonic peaks intensity in the power spectrum density plot.

To determine the D-banding from the selected collagen image, we recorded the maximum value in the 1st Harmonic peak in the PSD, corresponding to the D-periodic spacing along the average direction of the gap-overlap zone in Fig. 1. However, since the orientation of the fibrils within each image can vary (aligned side-by-side unidirectionally to randomly organized), we need to rotate the image to maximize the intensity of the first harmonic peak as we perform the 2D FFT. The D-banding corresponds to the distance between the fundamental peak and the first harmonic^33^. Thus, we have used the whole image to measure the D-banding (in case D-banding was visible throughout the image), or we have cropped the image for D-banding measurement to keep the area that had visible D-banding within that image (in case the D-banding was not visible throughout the image). The intensity of the first harmonic in the PSD was also maximized by rotating the image or cropping the image as we performed the PSD calculation. Finally, the D-banding was calculated by converting the pixel's index to periodic distances in nm. The distance in nm is calculated using Eq. (3):

3 $${\mathrm{v}}_{1\mathrm{st HArmonic}}=\frac{1}{\mathrm{N}{\Delta }_{\mathrm{x}}}=\frac{1}{{\mathrm{L}}_{\mathrm{x}}}$$

where ${\mathrm{v}}_{\mathrm{fund}}$ corresponds to the frequency of the first harmonic in the PSD (Hz), $\mathrm{N}$ corresponds to the size of the image (pixel), and ${\mathrm{L}}_{\mathrm{x}}$ corresponds to the distance (nm)^16,34,35^. The D-Banding periodicity values obtained using this PSD approach were compared with those calculated manually after plotting selected fibrils' long-axis profile using WSXM (5.0 Developed 10.2)^36^. For the manual calculation of the D-banding periodicity, ten fibrils were randomly selected from each AFM image.

### Advanced image analyses of collagen ultrastructure

All 5 × 5µm images (512 px) were segmented into 16 macro-pixel of 1.25 × 1.25µm (128px) using ImageJ software (version 1.53). Next, we perform the 2D FFT analysis for each macro-pixel. On each of the resultant 2D FFT images of the macro pixel, an orientation vector was orientated through the center of the two first harmonic arcs to establish the mean direction of the collagen fibrils in the area used to compute the 2D FFT. Phantoms AFM images of collagen fibrils were created with defined fibrillar alignment to control the level of structural anisotropy in the field of view. These phantoms were created from an AFM image by duplicating the same fibrils to form a stack of fibrils in different orientations using Image J. One singular collagen fiber, which displays a clear D-banding periodicity, was cropped to create the desired images. For example, if we wanted to obtain 2 populations of collagen fibrils oriented at 20° to each other, we would crop the fibril several times to create a population of fibrils. We then use this population of fibers twice, orienting them at 20° to each other with ImageJ. Once prepared, all images were processed using Fast Fourier Transform to generate the 2D FFT pattern. These patterns were subsequently analyzed using Image J to evaluate the correlation between the composition of the first harmonic arc and the phantom imaged.

### Evaluation of 2D FFT-based pathological anisotropy assessment.

To evaluate the use of the 2D FFT images (rather than their precursor AFM images) for pathological assessment, we performed a blind study using eight raters who did not have prior knowledge of AFM imaging or 2D FFT image interpretation. The assessors were first trained to recognize collagen fibril clarity, D-banding, orientation, and linearity on 3 AFM images and understand the impact of these four parameters on the respective 2D FFT images of control mice skin dermal collagen. We define a clear fibril as presenting obvious boundaries/edges with neighboring fibrils and a clear D-banding periodicity along its long axis. The same procedures were followed for 3 AFM images and their 2D FFT images of *Col1a1*^*Jrt/*+^ mice dermal collagen. After their training, the eight assessors presented 24 randomized 2D FFT images (12 from each mice group), which had been randomly selected from the entire set of images acquired. We performed reliability and repeatability tests using Fleiss's kappa score analysis. Inter-rater and intra-rater tests were performed, with the intra-rater repeated with a 7-day gap between each test. Fleiss's Kappa score was calculated to measure the concordance among all raters (more than two 'categorical' raters)^37^

For a scheme with n raters and K scoring categories, Fleiss calculates the proportion ${P}_{j}$ (Eq. 4) of all assignments, for all raters and all subjects, to each category j, for j = 1, 2, …, K, as follows:

4 $${P}_{j}=\frac{1}{N*n}\sum_{i=1}^{N}{n}_{ij}$$

where ${n}_{ij}$ is the number of raters who assign subject i to category j. The proportion of rater pairs who agree for subject i is now written as:

5 $${P}_{i}=\frac{1}{L}\sum_{j=1}^{K}{n}_{ij*({n}_{ij}-1)/2}$$

The proportion of rater pairs that agree over all raters and all subjects is defined as:

6 $${P}_{o}=\frac{1}{N}\sum_{i=1}^{N}{P}_{i}$$

Fleiss then estimates the probability of agreement 'by chance' as:

7 $$P_{e} = \mathop \sum \limits_{j = 1}^{K} P_{i}^{2}$$

Then, the Cohen's Kappa score was calculated by substituting these values of ${P}_{o}$ and ${P}_{e}$, into the Eq. 8. A coefficient of agreement for nominal scales. Educational and Psychological Measurement^38^:

8 $$K= \frac{{P}_{o}- {P}_{e}}{1- {P}_{e}}$$

Cohen's Kappa was interpreted as follows: 0.00–0.20: slight correlation"; 0.21–0.40: "fair correlation"; 0.41–0.60: "moderate correlation"; 0.61–0.80: "substantial correlation"; and > 0.80: "almost perfect correlation" as suggested by Landis & Koch^39^.

## Results and discussion

### Visual assessment of the collagen morphology

**Figure 2:**
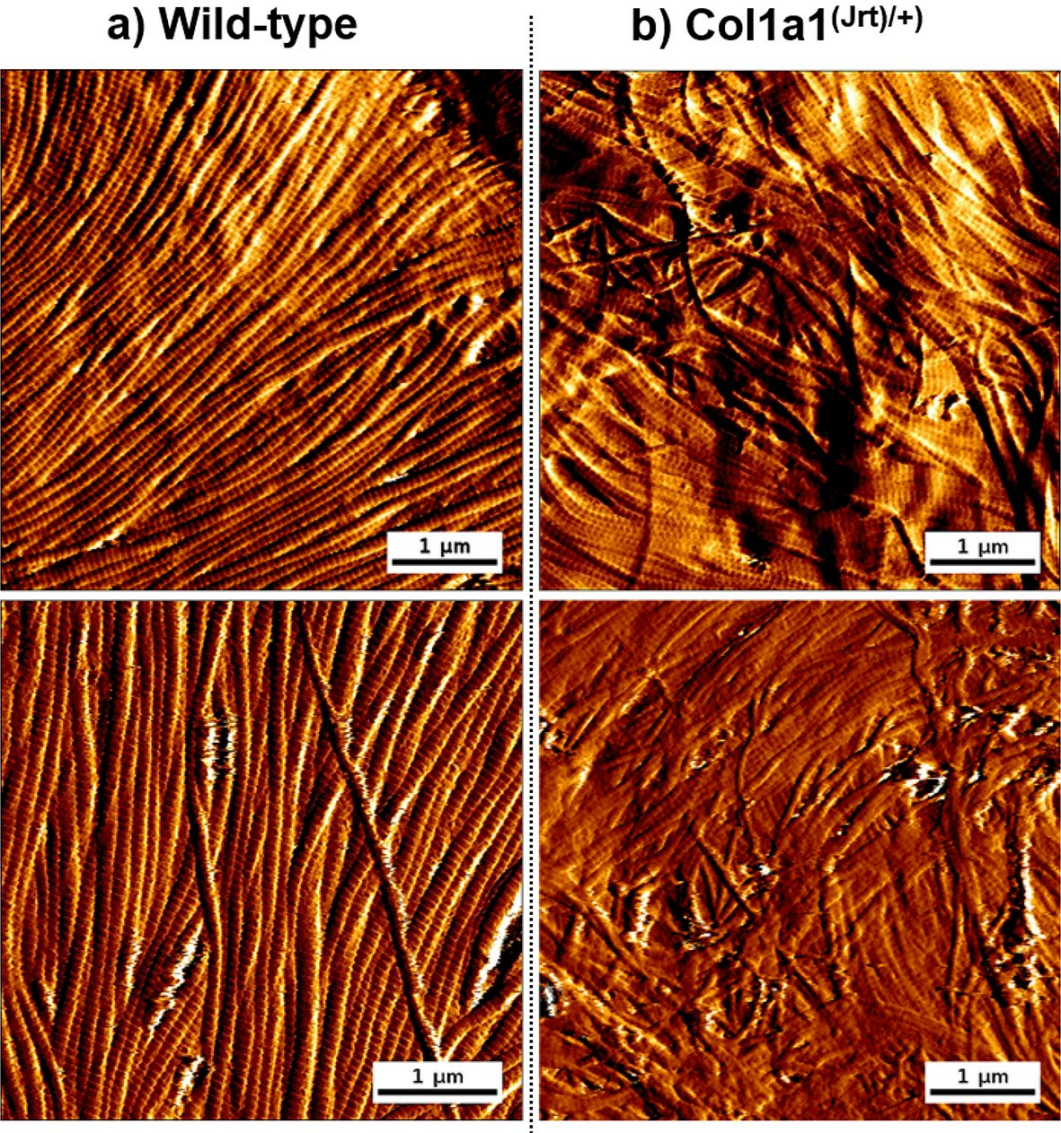
Representative AFM images of dermal collagen fibrils from (**a**) wild-type and (**b**) *Col1a1*^*Jrt/*+^ mice skin.

AFM images of the *Col1a1*^*Jrt/*+^ and wild-type mice skin are shown in Fig. 2. Collagen fibrils in the wild-type mice skin sections were visible with defined interfibrillar edges, D-banding periodicity was noticeable, and fibrils were straight within the image range and followed a primary direction.

In contrast, the collagen fibrils in *Col1a1*^*Jrt/*+^ mice had unusual kinks and turns; fibrils were not linear and randomly oriented, but D-banding was detectable. Overall, *Col1a1*^*Jrt/*+^ mice dermal collagen presented a significant level of morphological disorganization when compared to the wild-type mice. Although this mouse model was designed as a *COL1A1* mutation rather than *COL5A1* or *COL5A2* (predominantly expected in EDS), the skin clinical manifestations are reminiscent of those observed in EDS-affected patients, and these images recorded present the first account of the structural deformities associated with this condition at the nanoscale. It has been shown previously that the *Col1a1*^*Jrt/*+^ mice's skin had reduced tensile properties, the tail tendon appeared more frayed, and a third of the young adult mice developed kyphosis^40^. Here, we have studied the morphological changes at the collagen fibril level in *Col1a1*^*Jrt/*+^ mice skin for the first time, and demonstrated that *Col1a1*^*Jrt/*+^ mice skin exhibit significant morphological differences at the collagen fibril level. These differences were mainly pronounced in the collagen fibrils' clarity, orientation, and linearity.

### D-banding measurements using FFT and PSD

**Figure 3:**
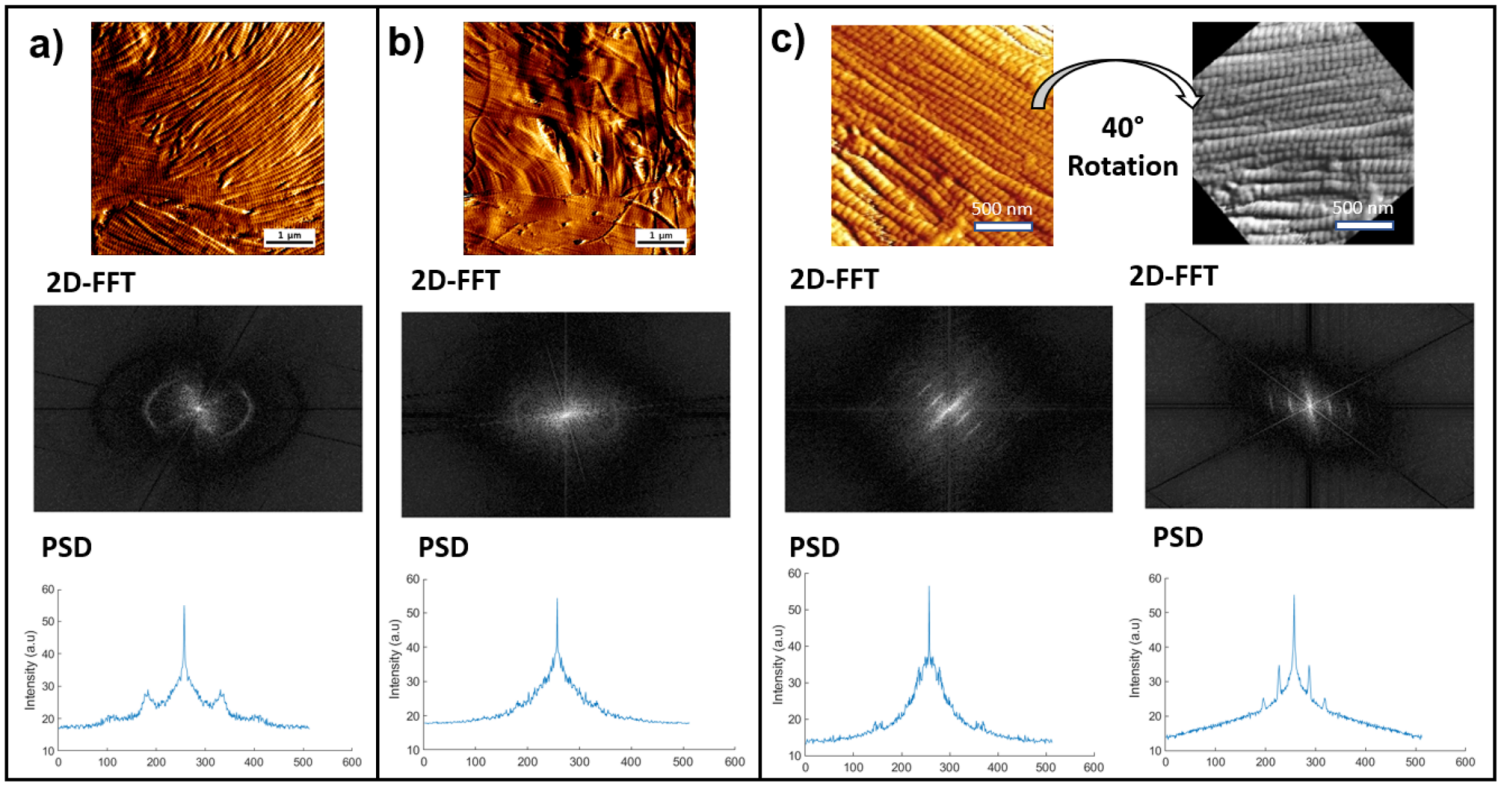
Representative AFM and 2D FFT images, PSD plots for (**a**) wildtype and (**b**) *Col1a1*^*Jrt/*+^ mice skin. (**c**) rotation of the original AFM image before the 2D FFT to maximize the amplitude of the first harmonic peak intensity.

In tissues such as skin, type I fibrillar collagen is readily identified by its repeating banding pattern, D-banding periodicity of 64-67nm along the fibril's length, arising from the careful staggering of collagen molecules (300 nm long, 1.5 nm wide)^41,42^. Measuring the D-banding periodicity has been carried out using multiple approaches involving Transmission Electron Microscopy^43–45^, Scanning Electron Microscopy^46–48^, or Atomic Force Microscopy^31,49–52^. While it is relatively trivial to measure the D-banding periodicity along a single isolated collagen fibril, it can be challenging to measure the average D-banding period over a broader area in which multiple fibrils are present, overlap and intersect. To facilitate this process, we developed a MATLAB code to automatically measure the average collagen D-banding of all fibrils in an AFM image and compare its outcome to the manual method. An example of this approach is presented in Fig. 3 and relies on optimizing the intensity of the first harmonic peak present in the PSD by rotating the image. The mean value obtained for the wild-type mice skin collagen was (64 ± 2) µm when measure computationally and (63.3 ± 1) µm when calculated manually. For *Col1a1*^*Jrt/*+^ mice (63.2 ± 1) µm was determined computationally and (63.5 ± 1)µm manually. Statistical analysis by one-way ANOVA of obtained values showed no significance between code employed or manually acquired. These measurements follow previous FFT and manual measurements for collagen type I D-banding^16,34,35,53,54^*.* It is, however, interesting to note that measuring the D-banding periodicity of ten fibrils manually yields the same D-banding value as that computed for the whole image. It is worth noting that the manual method requires the accurate placement of a line profile along the fibril long-axis, and baseline correction must be performed on the recorded profile along the long-axis of the fibrils to ensure the value of the calculated D-banding periodicity is not impacted by the fibril curvature. Finding flat fibrils may still be challenging, primarily when the measurement is performed on histological sections.

### Collagen isotropy through an AFM image

**Figure 4:**
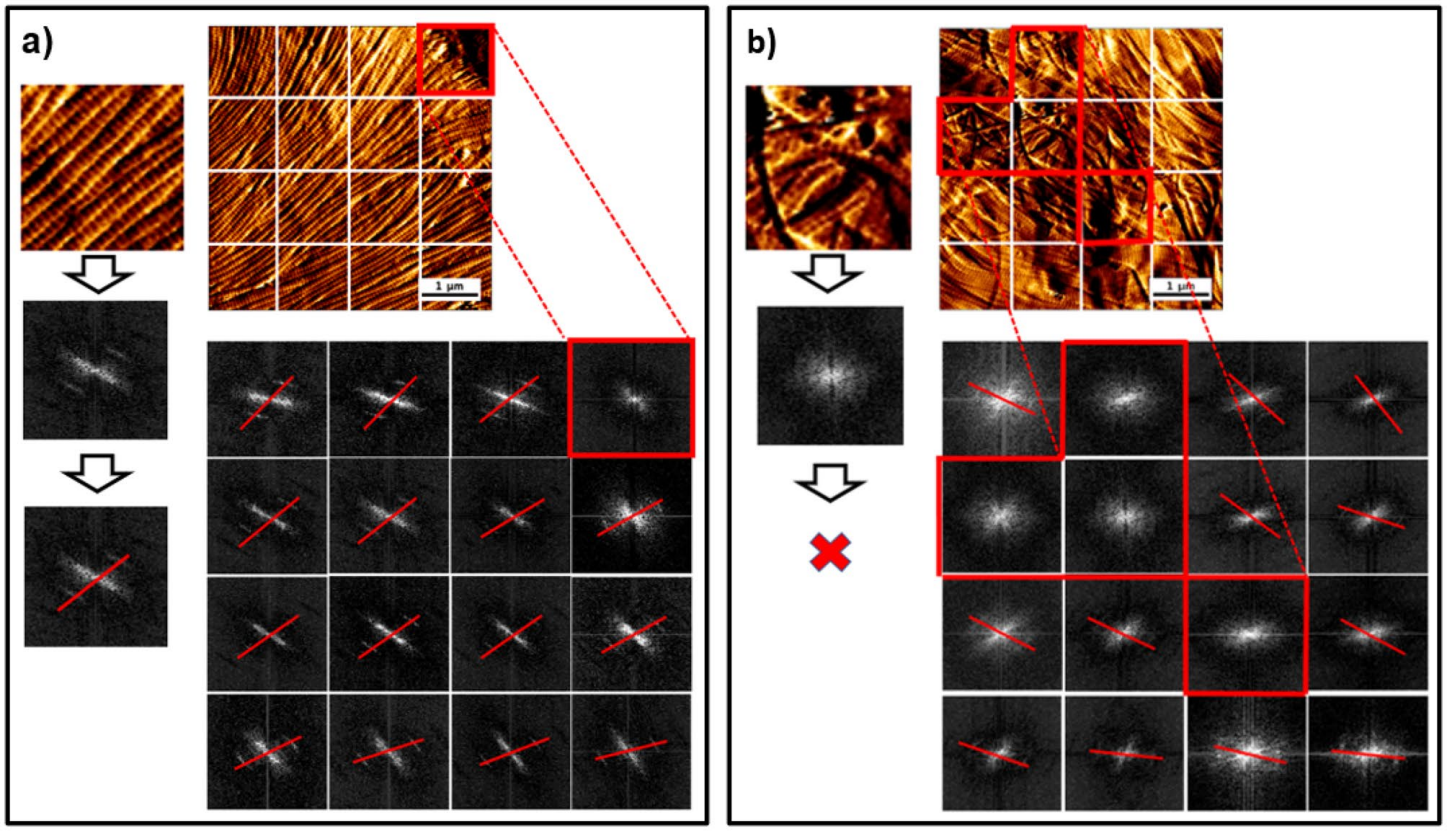
Segmentation of AFM image for 2D FTT image reconstruction performed for (**a**) wild-type and (**b**) *Col1a1*^*Jrt/*+^ mice skin. Images were segmented into 16 different 1.25 × 1.25um sections. An orientation vector (red line) was drawn through the center of the two first harmonic arcs to establish the mean direction of the collagen fibrils in the area used to compute the 2D FFT. No orientation vector could be plotted in areas where scatter rather than first harmonic arcs were visible.

The orientation of collagen fibrils within a tissue can vary depending on the tissue function or pathology. Tissues such as tendons or cornea exhibit well-ordered and aligned collagen fibrils that provide uniform tension. In the skin, we can find regions in which the collagen can also be highly organized (collagen sheet) and regions in which the collagen is more randomly organized, which can be associated with pathology such as skin fibrosis^19^. Within an AFM image, it can be challenging to visually assess the level of isotropy or orientation of the collagen fibrils. Therefore, we segmented the AFM images to alleviate this problem to study collagen isotropy. A preliminary study confirmed that we could not obtain sufficiently detailed 2D FFT of the macro-pixel if the macro-pixel contains less than 128 pixels. After transforming each macro-pixel in 2D-FFT, we reconstructed the 5 × 5µm with the 16 2D-FFT macro-pixel. Doing this enabled us to account for the local variation of the collagen isotropy within the image area recorded and simplified the interpretation of the collagen isotropy. As shown in Fig. 4a, wild-type mice skin presented mostly aligned and clearly defined collagen fibrils, ensuring a strong FFT first harmonic displayed a bright arc in the 2D FFT image. In contrast, *Col1a1*^*Jrt/*+^ mice's skin FFTs did not present any fundamental harmonic, leading to the display of a scatter rather than a bight arc in the 2D FFT image. We associate this "scatter" with the pathological anisotropy of the collagen fibrils, as shown in Fig. 4b. Additionally, as shown in Fig. 4a,b, collagen fibril orientation can be determined from arcs or scatters in the 2D FFT images, as explained in 2.4.2. Previously FFT studies have been used to study fibril alignment in electrospun model scaffolds comprised of straight fibrils with a fan-shaped pattern^55^. However, in our study, the segmentation of the AFM image allowed us to examine a whole AFM image that contained fibrils that were not entirely unidirectional. This method could be used to quantify collagen fibrils anisotropy associated with pathological conditions such as osteogenesis imperfecta, scurvy, and EDS.

### Collagen fibrils angular distribution

**Figure 5:**
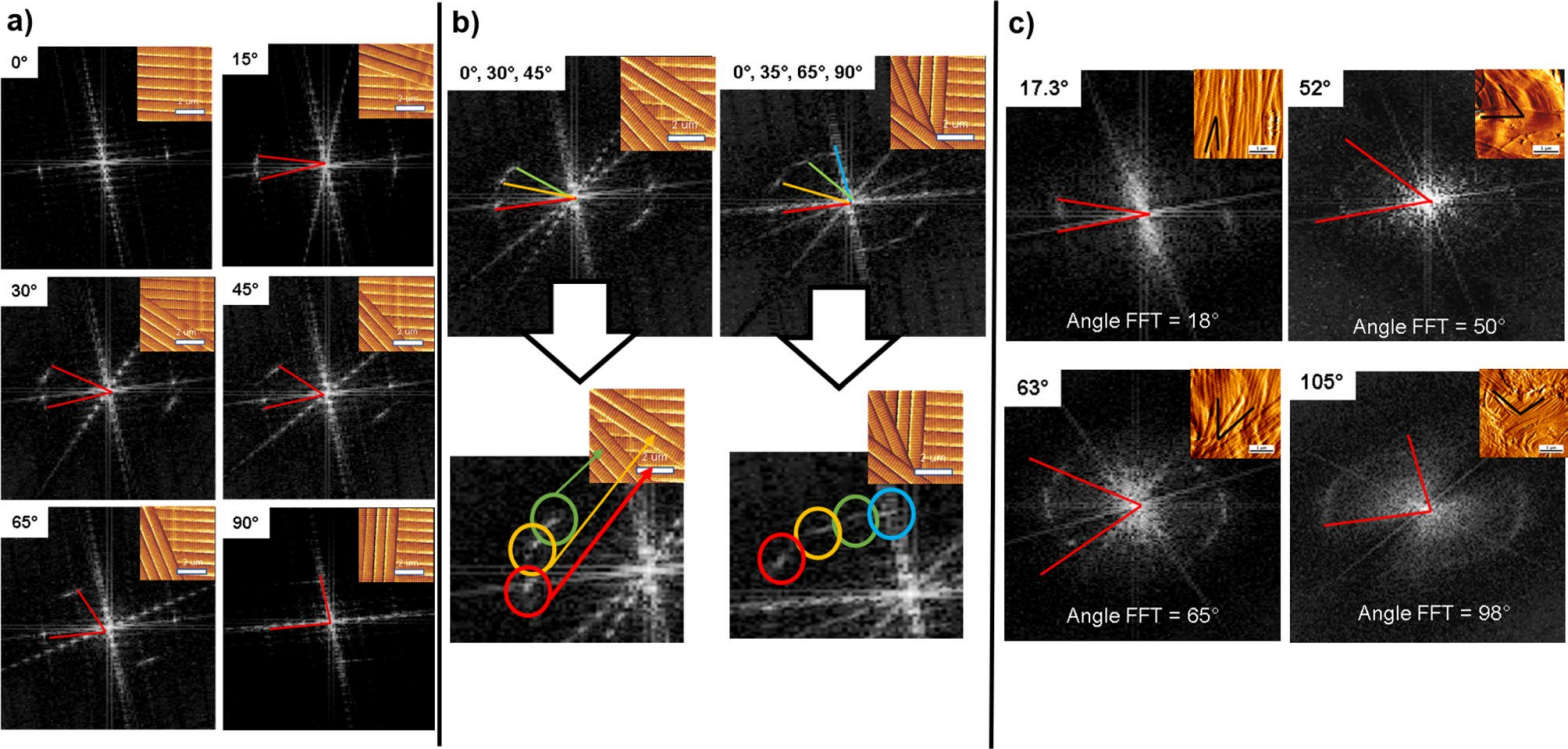
AFM image phantoms (**a**) & (**b**) and their respective 2D FFT images presenting discrete points corresponding to the two or more groups of fibrils in the original phantom image. (**c**) confirmation of the fibrils' angular distribution using AFM images of *Col1a1*^*Jrt/*+^ and wild-type dermal collagen.

When the first harmonic arcs can be readily seen on the 2D FFT image, plotting an orientation vector passing through the center of both arcs is possible. The angle of this orientation vector corresponds to the mean angular orientation of the collagen fibrils within that macro-pixel and of the entire AFM image. To confirm this in silico, we developed phantoms AFM images of collagen fibrils. Figure 5a presents images of collagen fibrils with one or two defined orientation(s) and their corresponding 2D FFTs. The 2D FFT only presents a single discrete point at the position of the first harmonic peak (rather than an arc or scatter) when the collagen fibrils are uniformly aligned (zero degrees deviation between groups of fibrils). When we added a second group of fibrils with a slight angular deviation, the 2D FFT image presented two discrete points corresponding to the two groups of fibrils in our phantom image. The angle between the two discrete points in the 2D FFT corresponded to the angle between the two groups of collagen fibrils present in the phantom AFM image. We increased the number of collagen fibril groups with each at a different angle, as demonstrated in Fig. 5b. When more than two groups of collagen were oriented in different directions, the 2D FFT presented discrete points associated with each of these groups. Still, the points started forming the first harmonic arc, as found when processing more complex collagen images^33^. For example (Fig. 5b), when the phantom image was made of fibrils oriented at 0°, 30°,45° and 60°, four discrete points were observed in the 2D FFT and formed an arc. The development of such phantoms confirmed that it is possible to measure the average angular distribution of collagen fibrils within an AFM image by performing an FFT and measuring the angle of the first harmonic arc in the 2D FFT. However, if the 2D FFT presents a scatter rather than an arc, it would be to measure the angular distribution of these fibrils.

Additionally, we have also found that the wideness of the arc changes depends directly on the direction of collagen fibrils. The wider the arc, the bigger the angle between collagen fibrils. It has also previously shown that the degree of anisotropy correlates with the arc aperture in the FFT, and this observation follows previous angular distribution studies^23,56^. We applied this method on randomly selected images recorded on the skin of the *Col1a1*^*Jrt/*+^ and wild-type mice, as presented in Fig. 5c. As expected, the arc formed in the 2D FFT image corresponds to the angle distribution of the fibrils within the AFM image. Computing the angular distribution of the fibrils directly on the AFM image by image J and comparing it to the arc aperture angle on the 2D FFT image yielded the same result for collagen fibrils anisotropy.

### 2D FFT as a tool to define a collagen-pathological phenotype

The interpretation of AFM images can be challenging for a non-AFM specialist, especially when such images contain repetitive structural features such as fibrils in straight or random orientation, presence or not of D-banding periodicity, for example. These features are common across all AFM images obtained on the skin. We previously described how 2D FFT could quantify structural parameters associated with collagen nanoscale topology^23^. We explore next whether the use of 2D FFT image interpretation by non-AFM specialists (i.e., the raters) could differentiate images obtained between wild-type and pathological collagen, as presented in Fig. 2. Following a short training, the raters were asked to classify a series of 2D FFT images as wild-type or pathological based on their appreciation of the 2D FFT images related to the presence of the first harmonic arc or a scatter. We performed reliability and repeatability tests using Fleiss's kappa score analysis. The inter-examiner assessment of 24 randomized 2D FFT images yielded K = 0.71, and the intra-examiner assessment yielded K = 0.66.

Both inter-rater and intra-raters’ tests showed substantial strength of agreement between the raters. It is accepted that a Kappa score over 0.7 is considered to be a very good agreement. In contrast, a Kappa score less than 0.6 shows inadequate agreement among the examiners, thus lacking confidence in the study results^57^. Based on this result, it is possible to differentiate between wild-type and pathological collagen by observing the 2D FFT images computed from the original AFM images obtained on these samples. Another study used a similar Kappa score to assess traumatic cerebrovascular injury detected on computer tomography angiography. Their method has been accepted and used in clinical studies^58^. While structural anisotropy in the dermal collagen network as a function of connective tissue disorders is conducive to such a study, it would greatly enhance the use of AFM nanometrology for disease diagnostics. In this study, four raters were clinicians without prior knowledge of AFM imaging. After reviewing the training set, raters were able to assess the 2D FFT images confidently. While computing the collagen network pathological anisotropy using AFM imaging may not be sufficient to diagnose the conditions, it would remain a valuable tool to appreciate in extent or severity of the condition, such as fibrosis throughout collagen-rich tissues. The success of such a computational approach still resides in the technical skills of the AFM user to acquire the best image possible. Considering the time required to acquire an AFM image (~ 3min), much effort needs to be made to increase the versatility of this approach for rapid assessment of tissue^59^.

## Conclusion

While collagen structure has been studied for decades at multiple scales, the interpretation of images such as those acquired by Atomic Force Microscopy can lead to complex interpretation due to collagen network pathology-driven anisotropy. Herein, we showcased a facile method to compute collagen network pathological anisotropy using AFM imaging based on 2D Fast-Fourier Transform. This approach was tested experimentally against wild-type and pathological dermal collagen and *in-silico* by creating phantoms AFM images of collagen fibrils with a different angle (Supplementary Information 1). Here we confirm that the presence of the first harmonic arc could be associated with the presence of well-defined and orientated fibrils in the AFM image. In contrast, the presence of scatter would indicate fibrils are poorly defined and randomly orientated. We also confirmed that the interpretation of the 2D FFT images could be used as a rapid approach to discriminate the dermis of wild-type and *Col1a1*^*Jrt/*+^ mice marking a first step towards using AFM image analysis for pathological assessment.

### Supplementary Information

Supplementary Information.

## Data Availability

The data and the code used in this current study are available from the corresponding author (LB) at a reasonable request.
