# Supplementary material for: Development of a facile method to compute collagen network pathological anisotropy using AFM imaging
Source: Sci Rep. 2023 Nov 17;13:20173. doi: 10.1038/s41598-023-47350-y (PMC10656449; doi:10.1038/s41598-023-47350-y)
Supplement: Supplementary file 1 — Supplementary Information. [file 41598_2023_47350_MOESM1_ESM.docx]

**Step-by-Step Guide to the Method**

1. 2mm-by-2mm dorsal skin sections were acquired from 14-week-old wildtype and Col1a1Jrt/+ mice.
2. Skin tissues were frozen.
3. Ten-micrometer sections were made longitudinally through the epidermis and papillary dermis.
4. 5x5 µm², 512x512-pixel AFM images were acquired on seven random areas directly on each slide using Nano-wizard 4 Bioscience (Bruker, Germany), operated in contact mode under ambient conditions, and with MSLN-10-C probes (Bruker, Germany). All AFM image analysis was performed on the deflection images recorded using JPK data processing software (version 6.3.5).

The following steps were done in MATLAB (version 9.12.0.1884302, The MathWorks, Inc., Natick, Massachusetts, United States) unless mentioned otherwise. The code will be available upon reasonable request from the corresponding author.

1. Two-dimensional Fast Fourier Transform (2D FFT) was performed for each AFM image.
2. The Fourier transform, when converted into a power spectrum without imaginary parts, generates the PSD.
3. D-banding was measured using the first harmonic peak in PSD. (Note: Occasionally, the image should have been rotated to achieve the maximum peak, and the code provides a feature for image rotation).
4. The D-banding was also measured manually using WSXM (version 5.0 Developed 10.2).
5. All 5x5 µm images (512 px) were segmented into 16 macro-pixels of 1.25 x 1.25 µm (128 px) using ImageJ software (version 1.53).
6. On each of the image segments made from step 9, a 2D FFT image was generated. An orientation vector was created through the center of the two first harmonic arcs to establish the mean direction of the collagen fibrils in the area used to compute the 2D FFT.
7. To confirm the results of step 10, phantom images were created from an AFM image by duplicating the same fibrils to form a stack of fibrils in known orientations using ImageJ.
8. To evaluate the use of the 2D FFT images for pathological assessment, Kappa analysis was performed with eight raters who did not have prior knowledge of AFM imaging or 2D FFT image interpretation.
